# The design of smart classroom for modern college English teaching under Internet of Things

**DOI:** 10.1371/journal.pone.0264176

**Published:** 2022-02-18

**Authors:** Ruihua Nai

**Affiliations:** College of Translation Studies, Xi’an Fanyi University, Xi’an, China; King Abdulaziz University, SAUDI ARABIA

## Abstract

This study aims to improve the efficiency of modern college English teaching. With interactive teaching as the core element, smart classrooms as technical support, and informationization, automation, and interaction as the main body, a smart system for college English teaching is established based on cloud computing and Internet of Things (IoT). The system is built using the B/S architecture and verified by specific example data, to prove the effectiveness of the proposed smart system for college English teaching based on the IoT. It is found that the smart platform for English teaching based on the IoT not only effectively improves the stability of the system, but also enhances the personal experience of students. The coordinated operation of the various modules reduces the response time of the system. When the number of users reaches 500, the average response time of the system is 3.65 seconds, and the memory and occupancy rate of the system are reduced. Students who receive smart classrooms for teaching have a greater improvement in the test results of various aspects of English without teacher intervention. The proposed model can significantly improve the performance of poor students and reduce the gap in learning performance in the class, which provides reliable research ideas for smart teaching in modern colleges and universities.

## Introduction

With the rapid development of new-generation information technologies such as cloud computing, Internet of Things (IoT), and artificial intelligence (AI), more and more colleges and universities are applying the latest information technology to the teaching. It not only enriches the teaching methods, but also meets the needs of students for diversified and personalized learning [1]. Education industry in China has entered the smart level in the information teaching after the emerge of the traditional teaching, audio-visual teaching, and digital teaching. As the main front of teaching, the construction of smart classrooms has become the core of the construction of smart campus [2]. The management of college teachers, especially the effective allocation of resources, has become a scientific issue of general concern from all walks of life with the expansion of college majors and the establishment of emerging majors [3]. The smart classroom aims to realize the diversified display of knowledge based on the existing information technology, so that students can fully absorb and understand the knowledge based on the informationized teaching experience. Based on the smart classroom, the system can realize the active dialogue with the students to ensure that the students can learn and develop autonomously, improving the efficiency of teaching process [4]. At present, there are many studies on building smart classroom network system and smart campus management system, which have been applied in practice [5]. Therefore, studying smart classrooms shows important reference value for building modern smart campus and promoting the transformation of talent training.

With the rapid development of IoT, how to enhance the management level of classrooms in colleges and universities and improve the utilization of classroom resources has become the core of the informatization construction of smart classrooms [6]. IoT is a network extended on the Internet, which realizes the interconnection and sharing of information with the help of local sensors, such as radio frequency identifiers and laser scanner systems [7]. IoT enables the high-speed information management, realizes resource sharing, strengthens the interaction between teachers and students, achieves more flexible deployment of college staff, and makes it easier to find shortcomings and difficulties in the teaching [8]. Due to the lack of interaction in current English teaching, the teaching process is boring, which seriously hinders the cultivation of English teaching talents [9]. Smart English teaching can promote teaching efficiency to a certain extent, enhance the interest of student, and meet the English learning needs [10]. However, there are currently few studies on the construction of smart classroom systems. How to use Internet of Things technology to build an efficient English learning management platform is of great significance to promote the development of English major students, so that both students and teachers in colleges and universities can easily and conveniently realize operation and management.

The innovation of this study lies in the application of the Internet of Things and cloud computing in the intelligent classroom system of college English to achieve efficient and interesting teaching. On this basis, the intelligent classroom information system is built and verified by specific performance tests and teaching examples. Through the analysis of the current situation of educational informatization, common problems in English teaching are found. The Internet of things system is applied to build a safe, reliable, and widely used intelligent English classroom comprehensive management system, and the English learning intelligent classroom system platform is proposed. Through the deployment of specific intelligent teaching system and analysis of practical teaching methods, reliable research ideas for intelligent English teaching are provided.

Five parts are arranged in this study. The introduction puts forward the importance of smart English classroom building and determines the main research ideas. The literature review analyzes the status quo of smart classrooms and teaching research, clarifies the existing problems in the current research, and determines appropriate research ideas. The third part introduces the research methods, clarifies the requirements of the design system, and describes the construction process of the system and the construction details of different models. The fourth part discusses the research results, analyzes the performance of the proposed model and the actual teaching effect, and compares the proposed system with other systems. The conclusion part introduces the conclusion, actual contributions, limitations, and future prospects of this study.

## Related works

### Smart learning

The core of smart teaching is breaking the traditional teaching scene and integrating all aspects of the teaching process to realize the interaction between teachers and students in the current teaching scene [11]. The current research on smart teaching mainly focuses on diversified teaching methods. Saunders et al. (2017) used the flipped classroom teaching method to change the traditional teaching method and add a variety of interactive processes. Through specific example data, it was discovered that the novel smart teaching method shows reliable applicability [12]. Xu et al. (2019) established an online teaching platform based on four advocacy modes (government-led, university student funding, teacher discipline, and academic guidance), and found that the improved system algorithm showed small errors compared with the latest research algorithm [13]. Biwer et al. (2020) verified the effectiveness of the system through the effective implementation of the smart teaching process [14]. Xu et al. (2020) proposed an intelligent training and education optimization network management model based on the big data mining, and it was found that this method can realize favorable resource scheduling and high degree information fusion [15]. Han and Xu (2021) proposed an intelligent education system, which realizes effective recommendation of courses and adjusts the learning ecosystem according to the interests of students, meeting the learning needs of college students [16].

### Smart classroom

As a typical intelligent learning environment, the smart classroom is an inherent requirement for the development of school informatization to a certain stage [17]. The exploration of the smart classroom teaching system is mainly based on the design concept of the smart classroom and the construction method of the teaching mode. It focuses on the transformation of the intelligent hardware platform of the classroom [18]. For the management and application of smart classrooms, MacLeod et al. (2018) analyzed the current teaching difficulties faced by multimedia classrooms and online classrooms, and made full use of technologies such as sensing, artificial intelligence, and the Internet to reconstruct the classroom environment and build new smart classroom [19]. Huang et al. (2019) gave the concept of smart classroom and the intelligent model of system-level smart classroom, and proposed an architecture for constructing and operating context-aware smart classrooms. The proposed system structure was feasible for building a situation-aware smart classroom in a smart campus [20]. Dao-en (2020) studied the development status of smart classroom in China and other foreign countries, compared the theories, designs, applications, and evaluations of smart classrooms applied in different countries, and found that the existing smart classroom can no longer meet the diverse teaching needs of students [21]. Kwet and Prinsloo (2020) found that the physical environment of smart classroom can promote teaching experience, technical application, data management, space environment, and technical environment [22].

### Summary

Through the analysis of recent relevant studies, it is found that although some progress and breakthroughs have been made in the existing classroom research on intelligent teaching, the current research on intelligent teaching mainly focuses on the innovation of teaching methods and the development of teaching resources, such as the application of flipped classroom, teaching word management, and information fusion. In addition, most of the existing studies on intelligent teaching and intelligent classroom remain at the theoretical level. In intelligent teaching, most studies focus on the display and presentation of information methods, but ignore the acceptance ability of students. The intelligent management of intelligent classes in colleges and universities combined with Internet of Things and the expansion of traditional classes are conducive to the efficient and convenient realization of all teaching processes and the improvement of teaching quality and management level. Therefore, it is feasible on the theoretical level, but how to build the corresponding intelligent teaching platform system according to the actual subject needs has become a scientific problem to be solved urgently in this field. Based on the analysis of the existing research, this work combines the Internet of Things with cloud computing and applies it to college English classroom, then in-depth analysis of the teaching characteristics of English classroom is carried out. It also proposes an intelligent teaching system platform for English learning, which provides a new teaching tool for college English classroom teaching.

## Research methodology

This manuscript has been approved by the school ethics committee. As the manuscript does not need ethical review. The approval document has been uploaded to the attachment as "other".

### Various system demands

**Fig 1:**
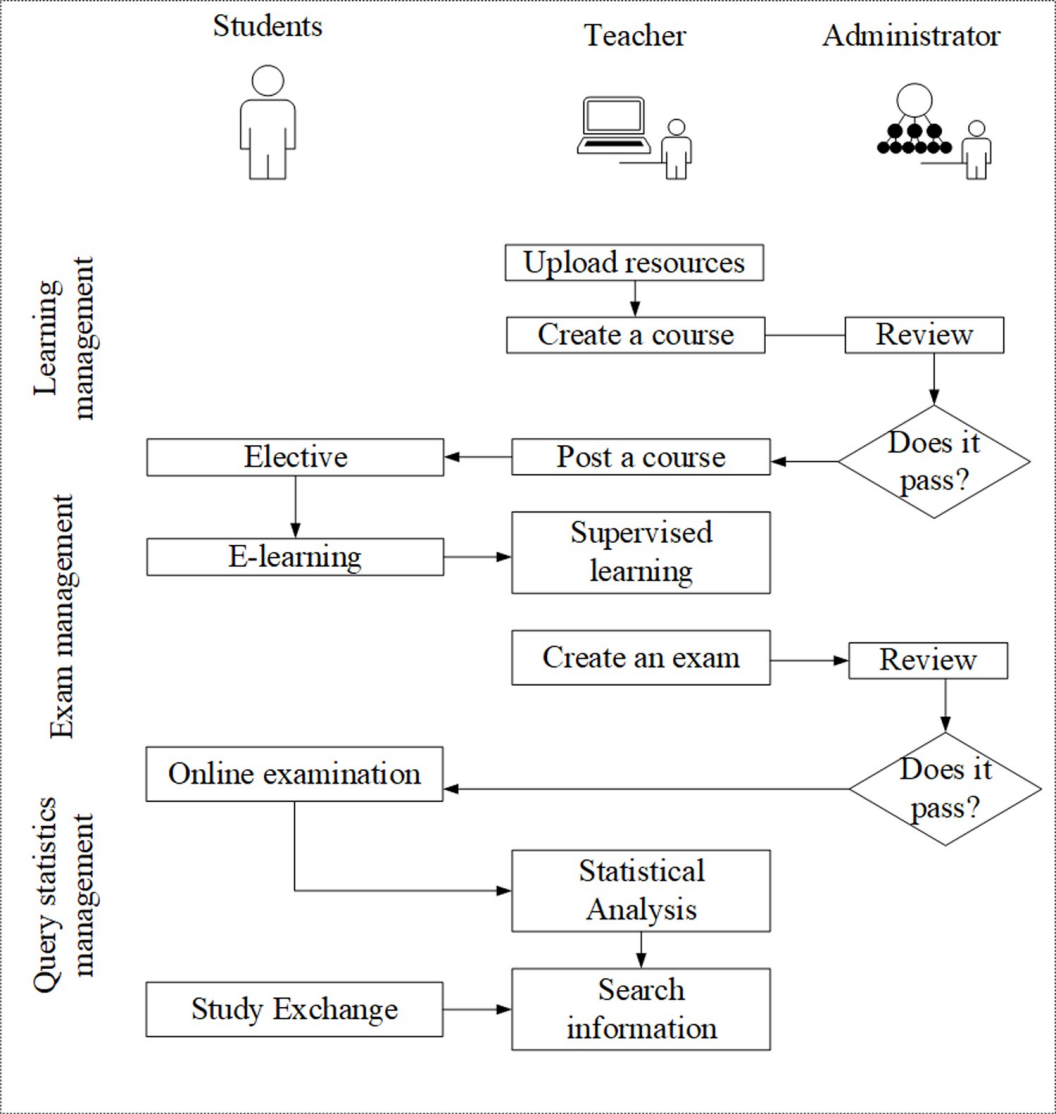
The process of system construction requirements of all parties.

I. Functional demands of the system are analyzed as follows. The needs of different individuals are analyzed firstly based on previous research combined with specific actual teaching, mainly covering the needs of students, teachers, and system administrators. The purpose is helping students to choose courses, learn online, complete exams, and communicate with each other. Moreover, it is necessary for teachers to upload learning materials, create learning courses, publish courses, supervise student learning, create exams, take statistical analysis, and query. Therefore, effective audits of different processes are required for system administrators [23]. The specific management and operation process is shown in Fig 1.

**Fig 2:**
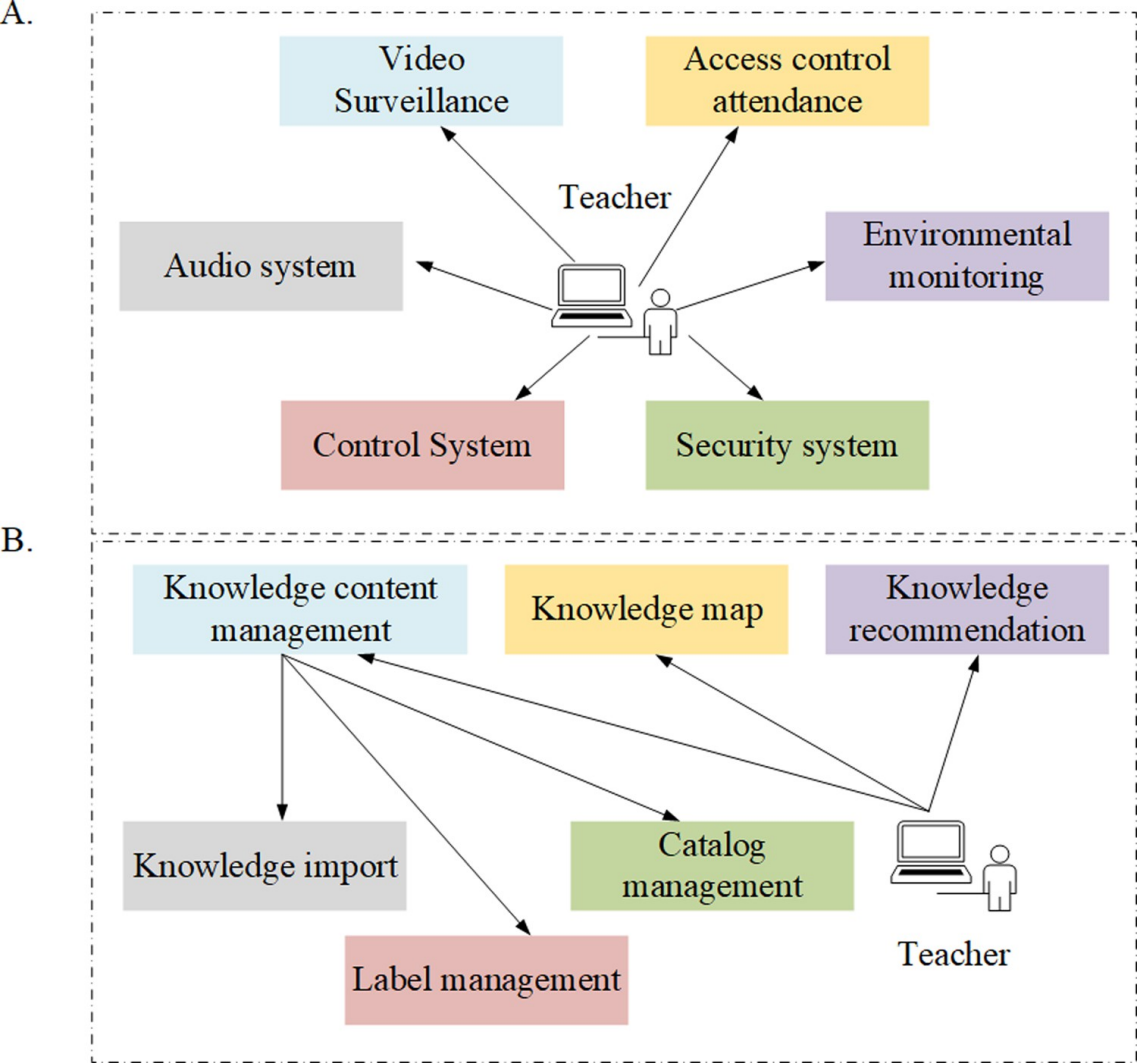
Analysis of teacher equipment and content management module.

For teachers, smart classrooms should cover six modules in terms of equipment management: video surveillance, access control and attendance, environmental monitoring, control system, security system, and audio system (Fig 2A). In the intelligent classroom teaching system, it is necessary to register the detailed file information of all equipment assets, collect the equipment information data reflecting the real-time status of the field, and remotely control and operate the field equipment system. The content management, knowledge map, and knowledge recommendation have to be ensured in terms of knowledge content (Fig 2B).

**Fig 3:**
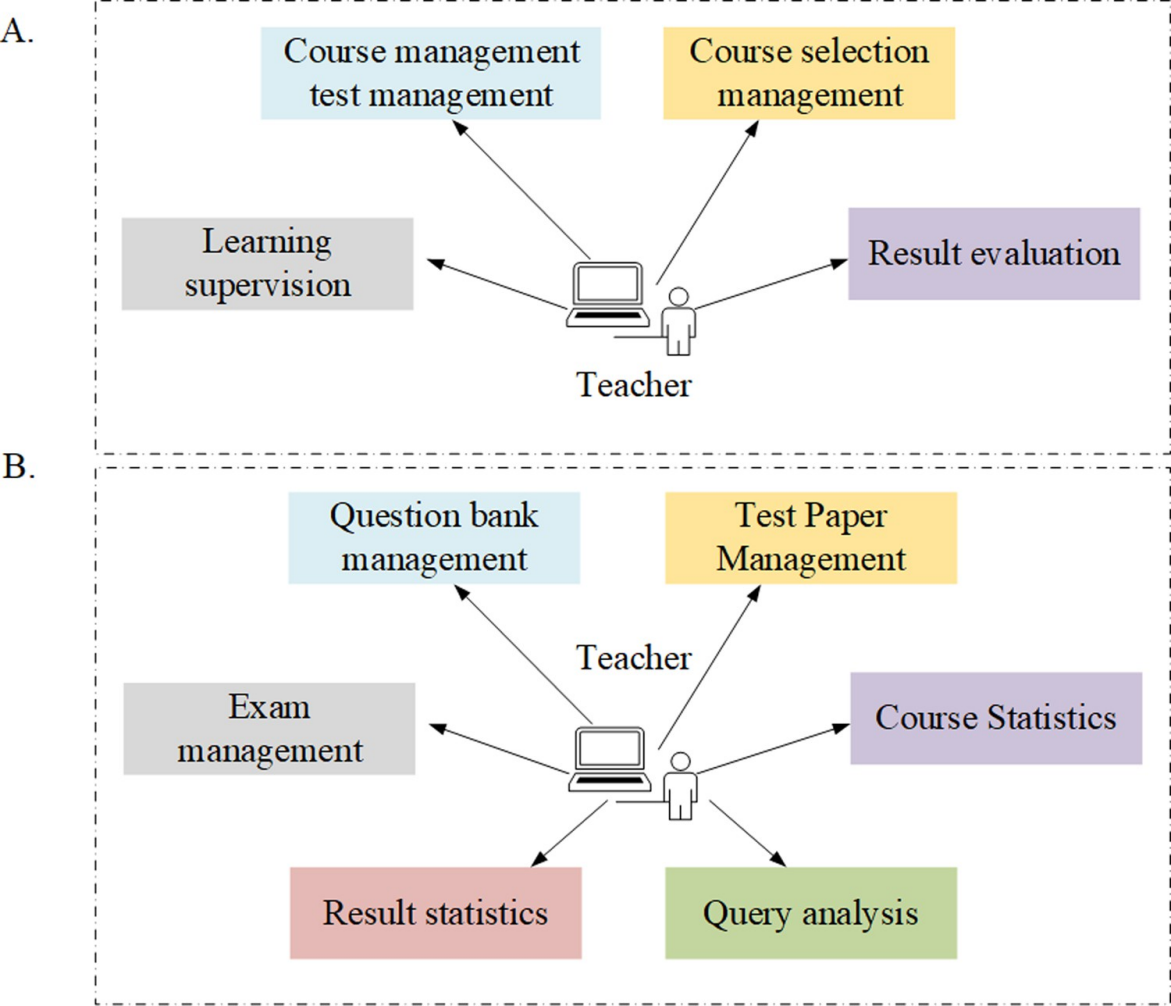
Analysis of teacher learning and examination management module.

Learning management aims to control the course selection of students, monitor their learning process, and evaluate their learning results. Therefore, it has to cover five parts of course, test, course selection management, learning supervision, and result evaluation (Fig 3A). Teacher administrators can manage the question bank by importing / adding / editing / deleting the test questions, arrange the schedule course exams, coordinate invigilators, organize exams, create and publish test papers, query analysis, take course statistics, review the test papers of students for scoring, and publish the test results. The details are shown in Fig 3B.

**Fig 4:**
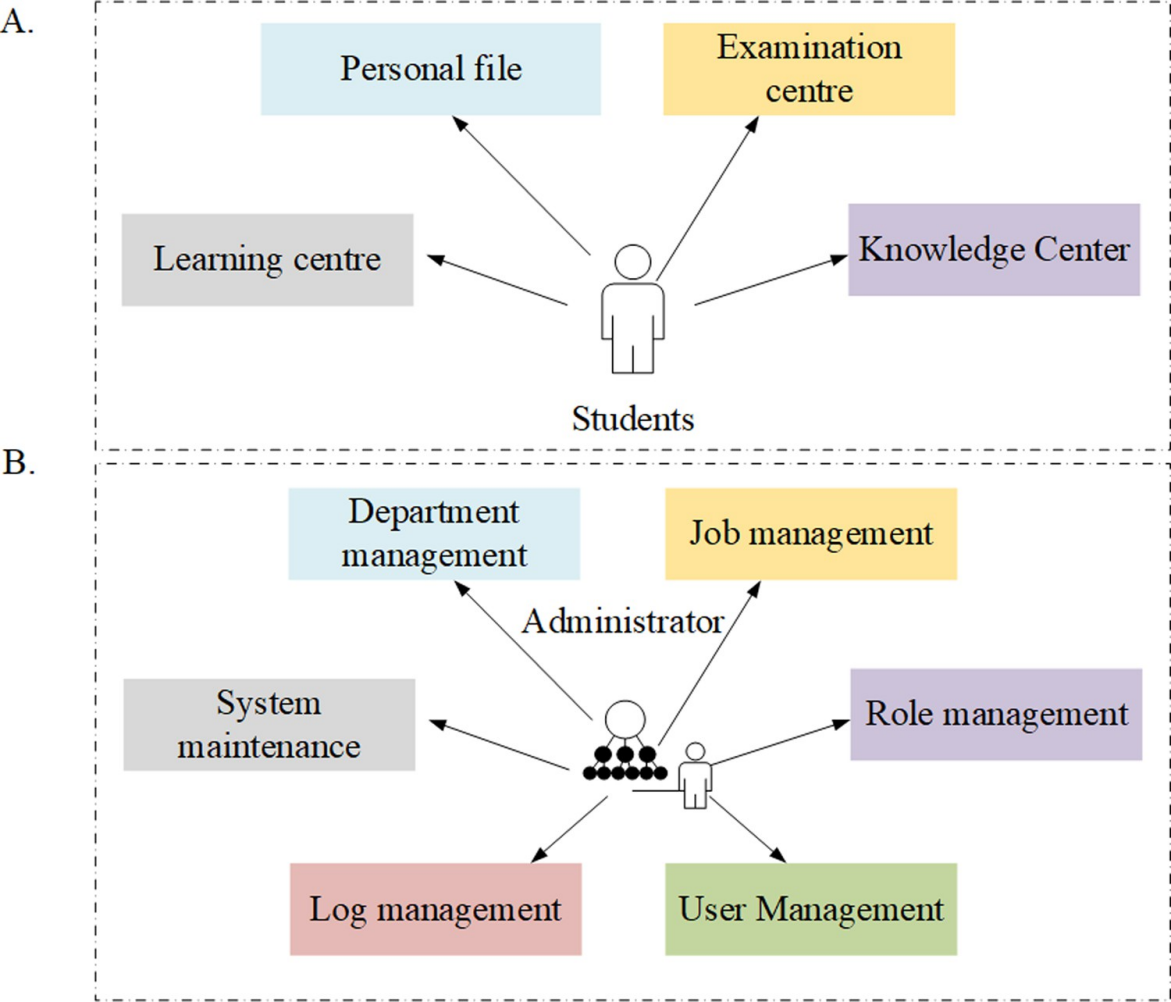
Analysis of students and system management module.

Learning, examination, knowledge center, and personal files constitute the functions of student center for students (Fig 4A). With this system, students can view the study plan, registrable exam, credits, course learning situation, relevant records of online exams, correct answers, mock tests and courses, and comprehensive exams. In addition, students can download / browse the knowledge resources to realize online learning. Department management, position management, role management, user management, log management, and system maintenance constitute the management functions for all users of the system (Fig 4B).

II. The performance requirements of the system are described as follows. Since the smart classroom teaching system involves the use of multi-level users of school teachers and students, it is necessary to set the maximum user load to 500 people. Due to the high real-time requirements of the system, the average response time of system transactions under the maximum user load can’t be greater than five seconds, the maximum response time can’t be longer than ten seconds, the success rate of transaction response should be greater than 98%, and the occupancy rates of central processing unit (CPU) and memory should be less than 80% [24, 25].

### Design of smart classroom system

The overall structure of the platform of the smart classroom system for English learning is supposed to be designed from three aspects of overall structure, main end structure, and sub-end structure.

**Fig 5:**
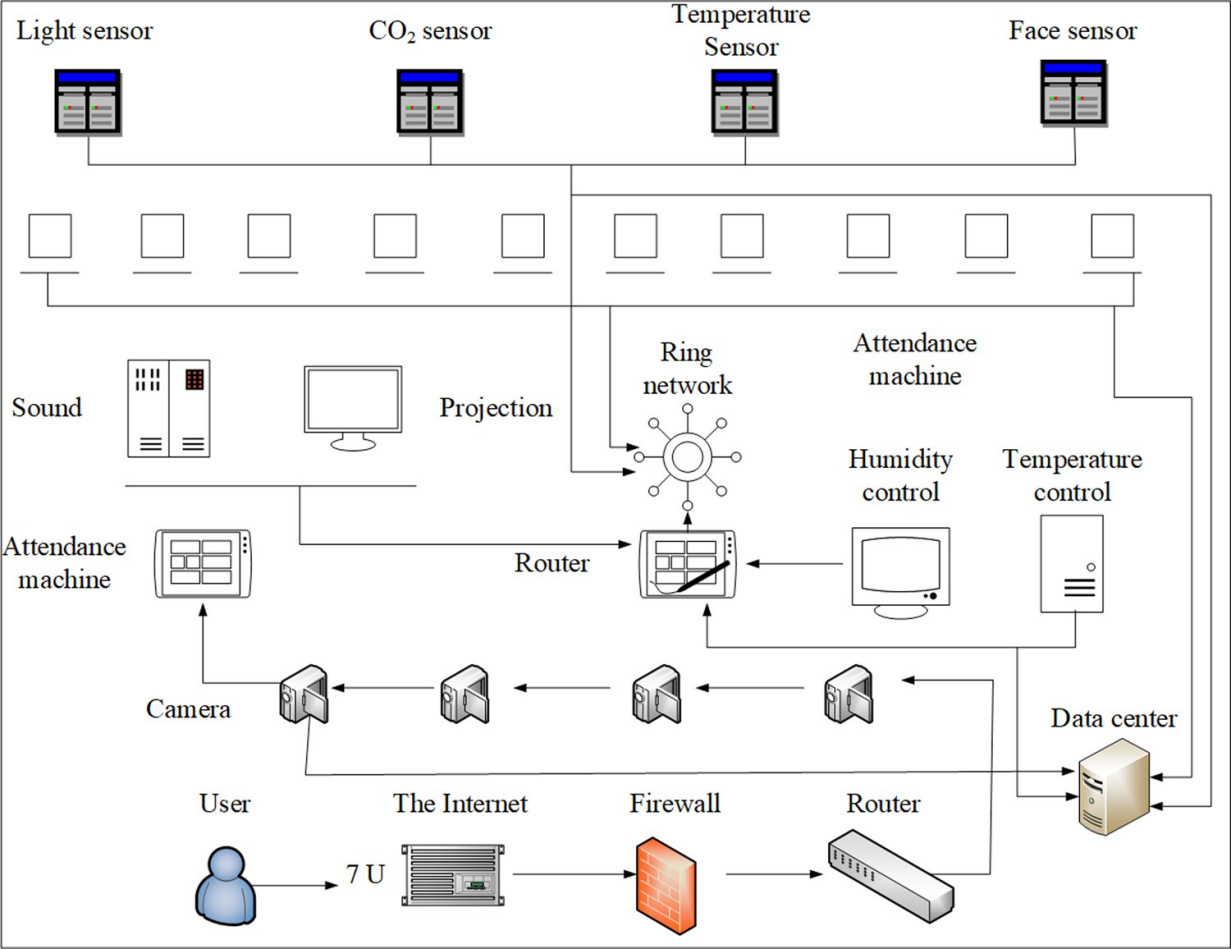
The overall structure of smart classroom.

The system is a comprehensive IoT sharing platform constructed based on the wireless switch, supplemented by the intelligent access gateway and its distributed wired system. Regarding the specific performance characteristics and applications of the smart classroom field equipment system, all equipment systems are connected through standard wireless module equipment or wired through smart access gateway to the integrated platform of smart classroom teaching system. In this way, it realizes the centralized and unified management of the system equipment through the server (Fig 5).

**Fig 6:**
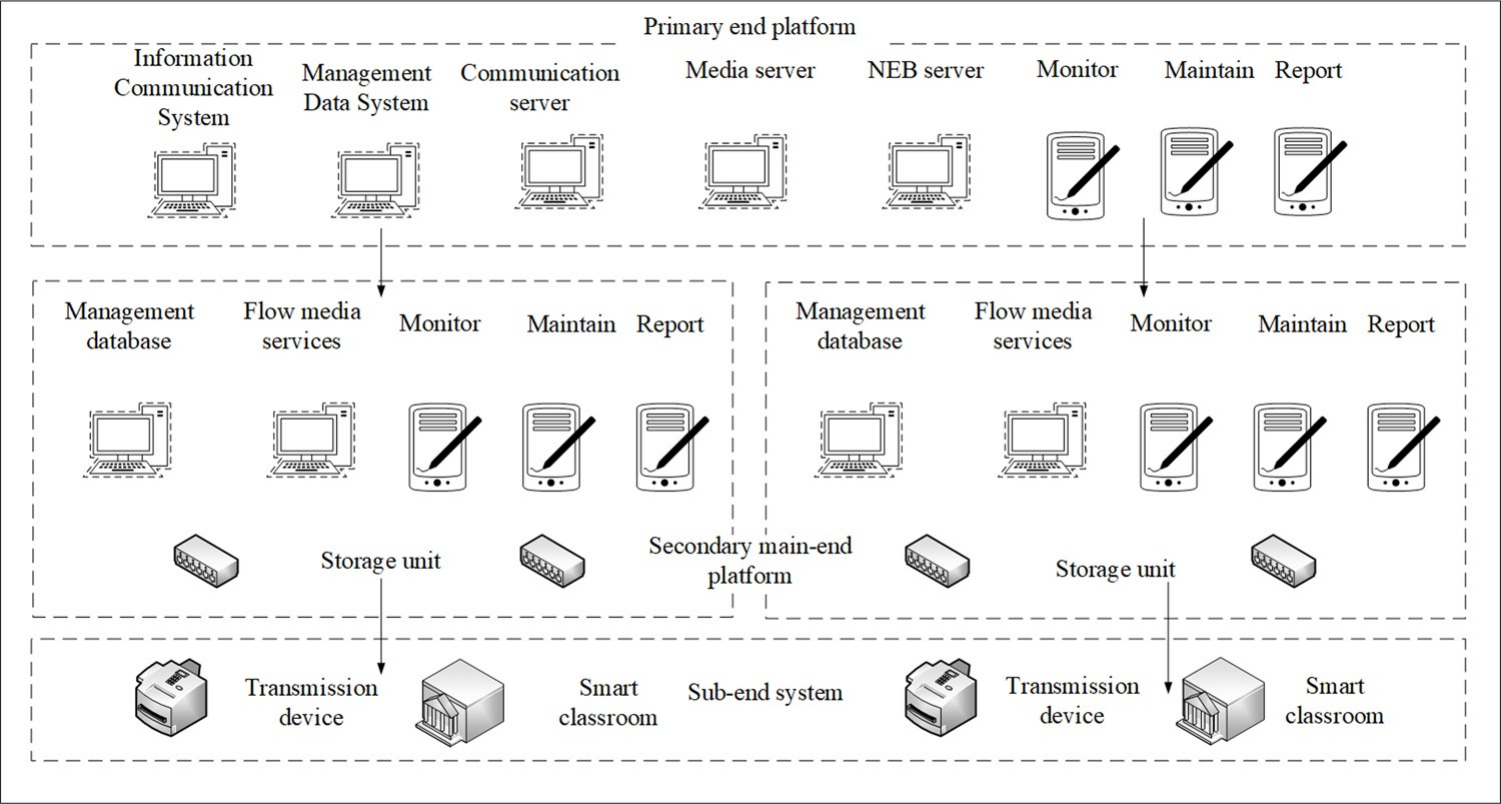
The overall topology of the integrated platform of the smart classroom teaching system.

Fig 6 shows that the integrated platform of the smart classroom teaching system is constructed in a cascading stack. The bottom layer of the comprehensive platform is the sub-end platform of each smart classroom. The middle layer is the secondary main-end platform that can be organized and organized according to the teaching subjects of the school. The top layer is the primary-end platform of the school level.

**Fig 7:**
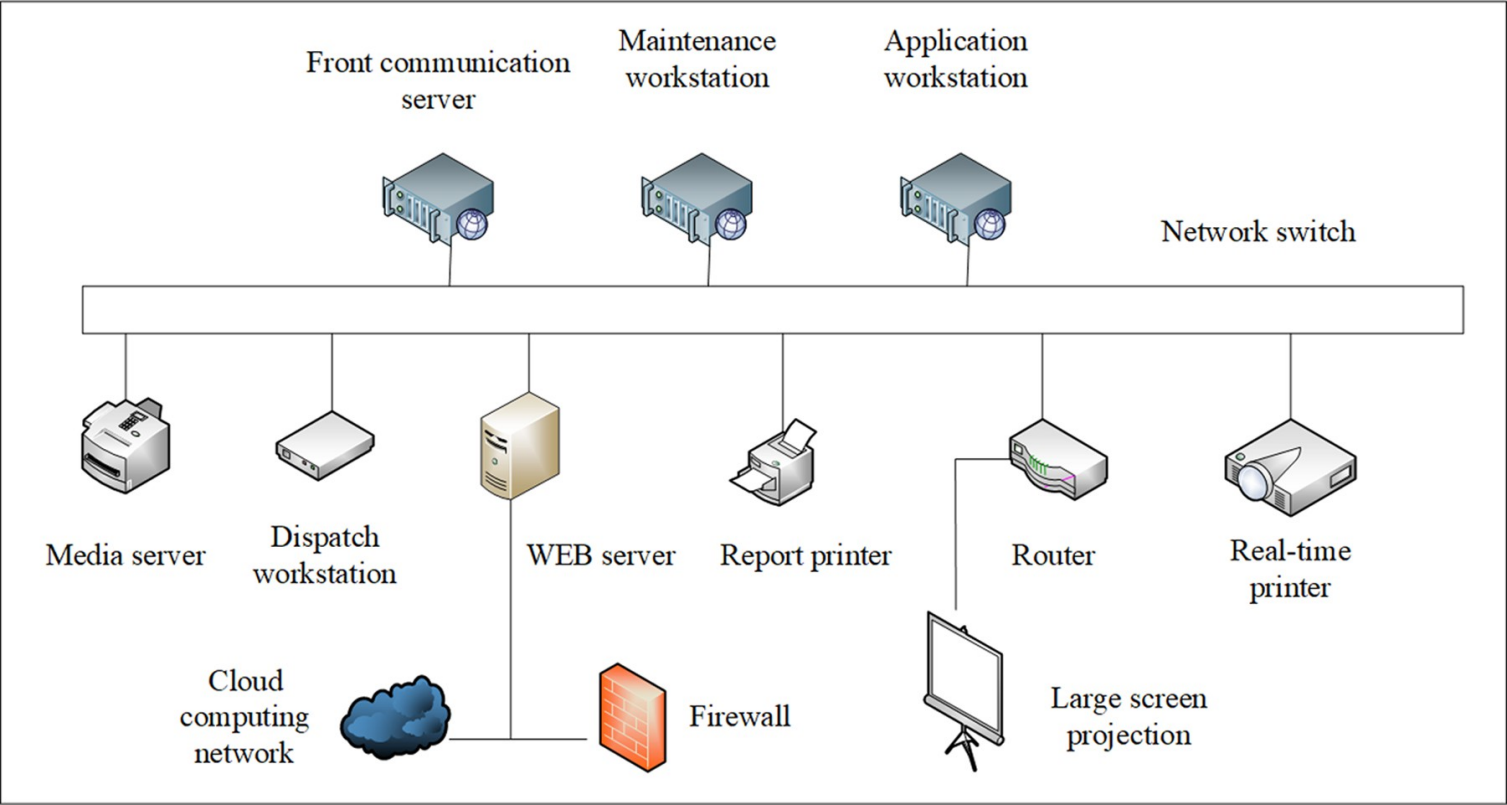
The main-end topology of the integrated platform of the smart classroom teaching system.

The main end of the integrated platform of the smart classroom teaching system is constructed by the dispatch, maintenance, application workstation, and the front communication, database, streaming media, and Web server. It is constructed through the network switch connection. The main end of the integrated platform communicates with the sub-end systems of each smart classroom through a standard communication protocol by the front communication server, and accesses the information data of each device subsystem in the smart classroom. The specific structure is shown in Fig 7.

**Fig 8:**
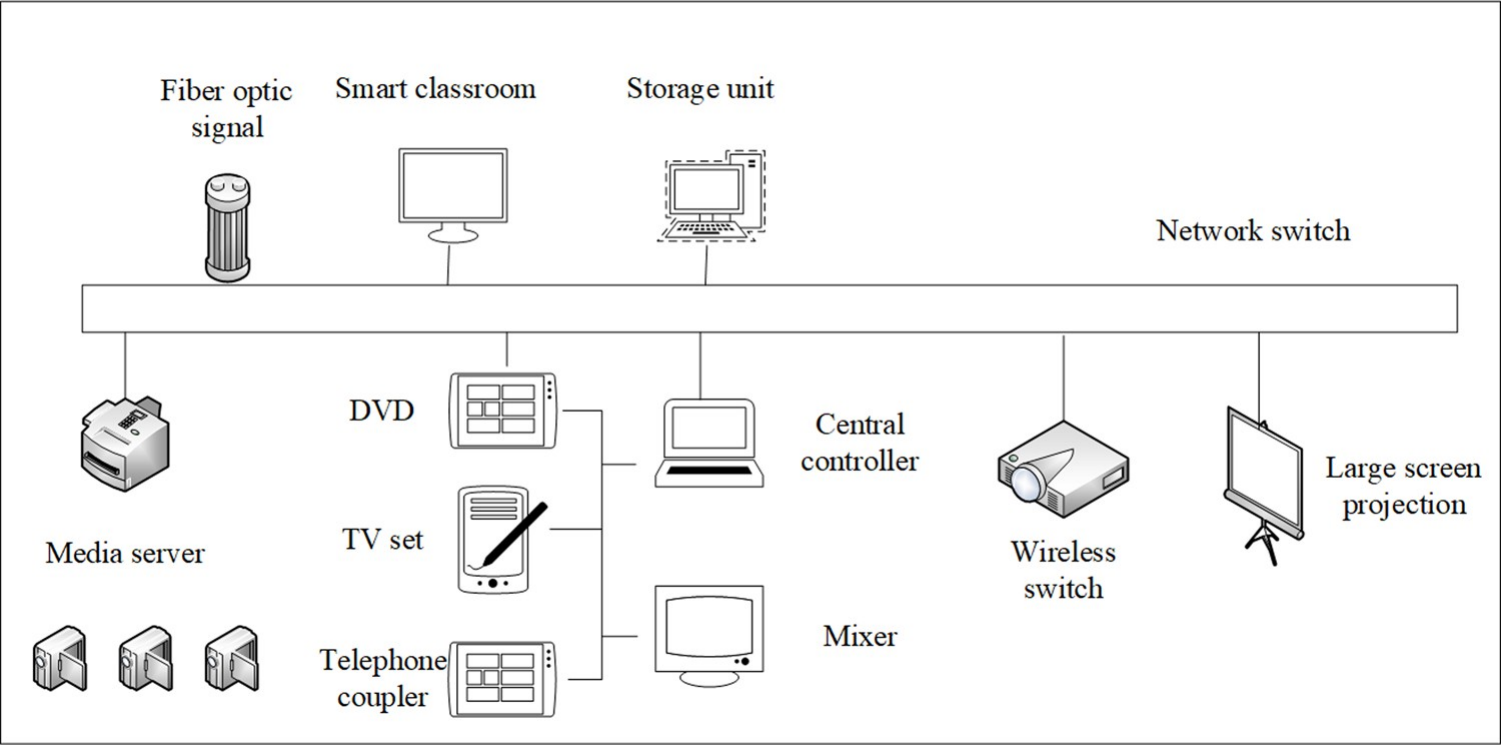
Sub-end topology of the integrated platform of smart classroom teaching system.

The sub-end of the smart classroom teaching system integrated platform is composed of wireless switch, smart access gateway, audio and video system equipment, and sub-end platform software. The wireless switch is connected to the device terminal with WiFi access, and the smart access gateway uses various interfaces except wireless to connect to the device terminal that requires wired access to complete various data collection and output control on the device subsystem of the smart classroom. The specific structure is shown in Fig 8.

### Implementation of smart classroom system

**Fig 9:**
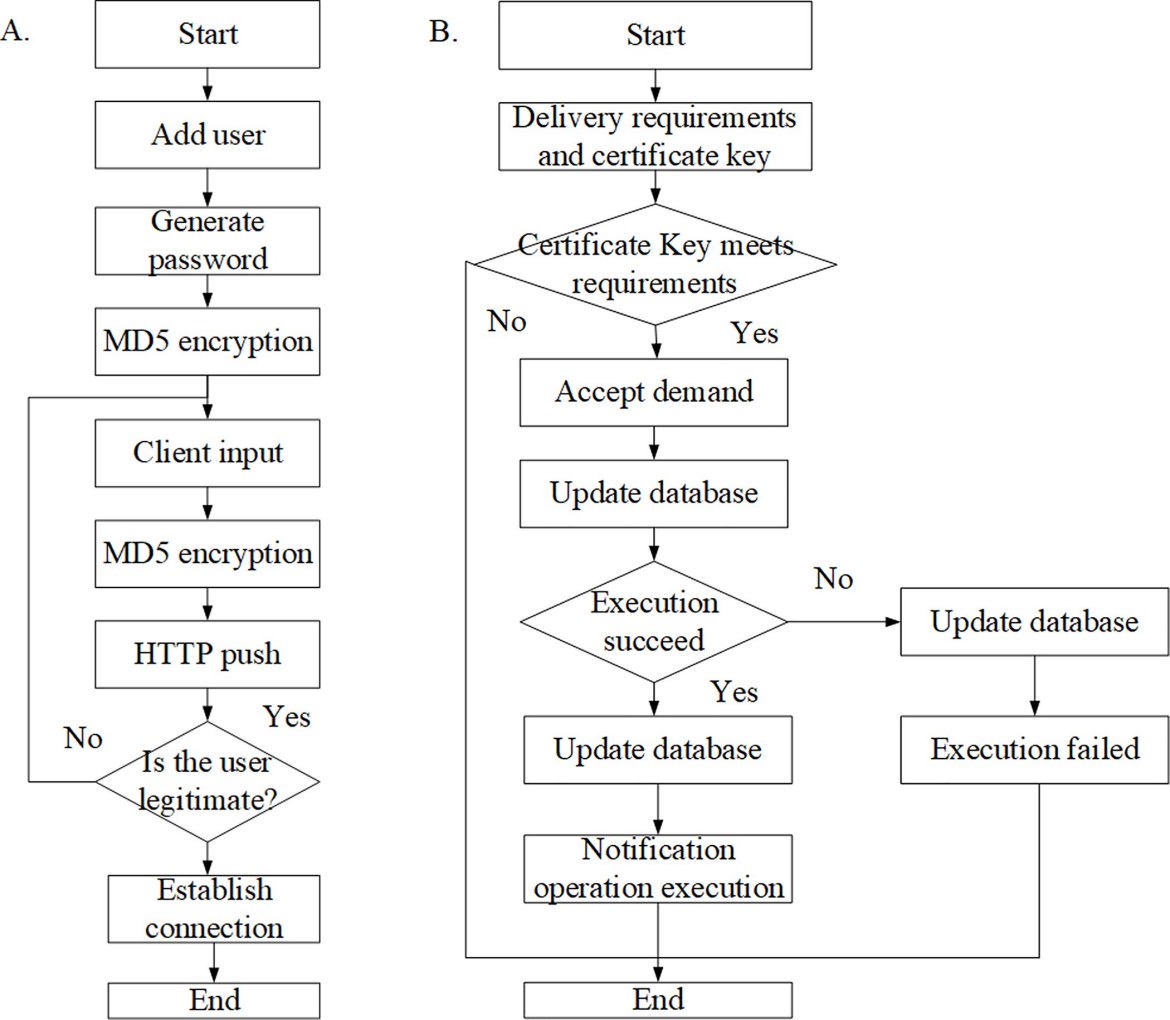
Connection between server and client.

A connection between the server and the client is established first. The user’s name and password are generated on the platform, which are entered by user and then passed to the server via hypertext transfer protocol (HTTP) protocol to verify whether they are legal. During transmission, the user’s name and password may be disclosed, so they are encrypted by MD5 encryption and stored in the server-side database. When the user name and password are successfully verified by the server, the connection between the server and the client can be successfully established [26]. The process is shown in Fig 9A.

The second step is the realization of client functions. The database record pushed by the server is received by an application on the Web client. The command is obtained using the get under HTTP [27]. After the database record is received, the value of the command will be stored in the corresponding field in the MySQL database. After the operation is successful, there will be a Notification status bar to remind the user that the operation is complete. The process is shown in Fig 9B.

**Fig 10:**
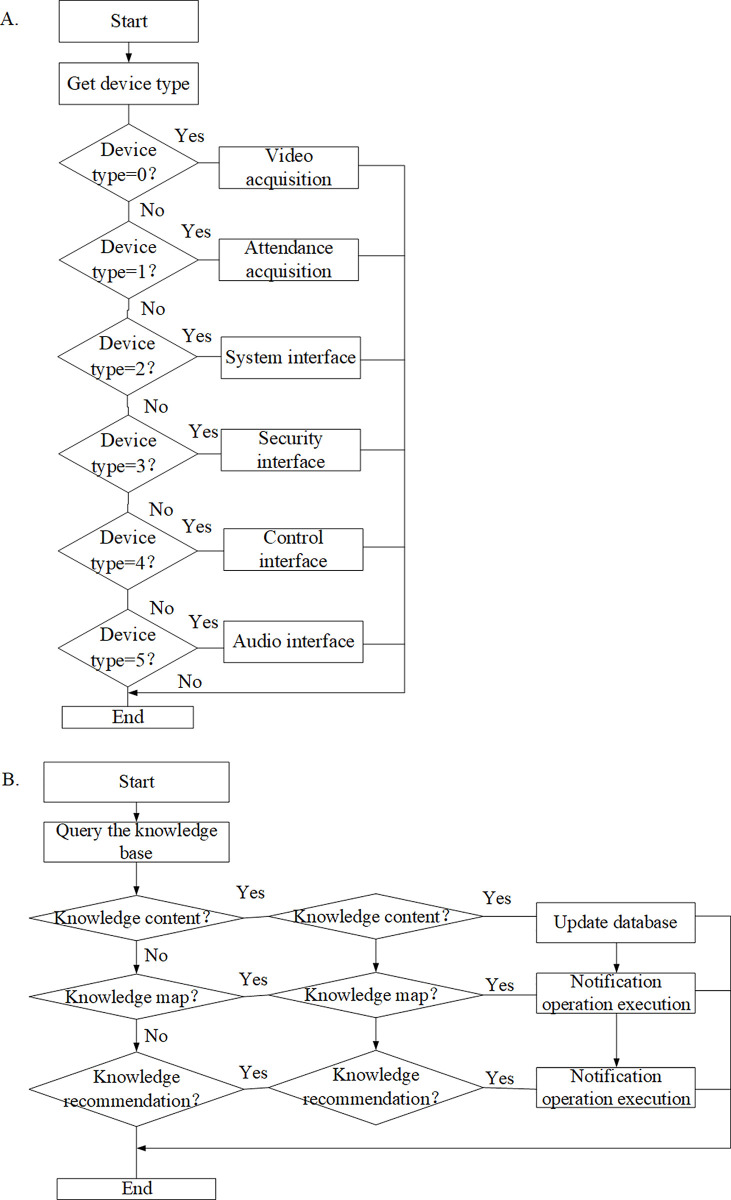
Flow chart of equipment list and knowledge management module.

The third step is implementing the device management module. The program uses the GetDeviceId() method to obtain the device identity (ID) selected by the user. The server queries the device information table according to the device ID, and returns the status information of the device system. The interface of the device subsystem selected by the user is presented, managed, and retrieved by InformationManager under the ProvisionDeviceInfo. The device can be edited using the EditDevice() and deleted by DelDevice(). The specific process is given in Fig 10A.

Then, the knowledge management module is implemented. The program queries the knowledge base information table according to the obtained knowledge base ID, and gives feedback as knowledge_code, knowledge_type, catalogue, and label. The specific process is illustrated in Fig 10B.

**Fig 11:**
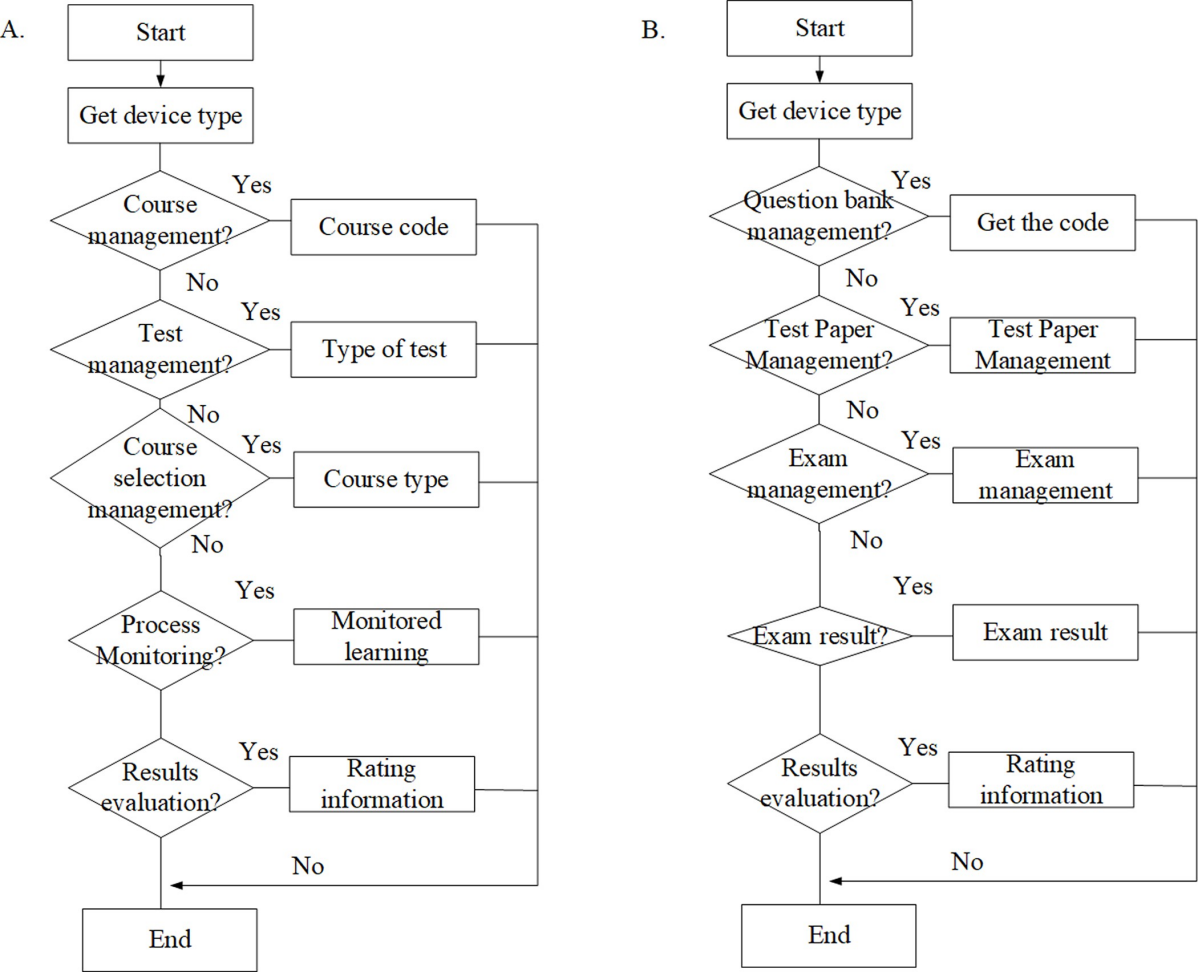
The flow chart of the learning management and exam management module. Note: Figure a shows the flow chart of learning management module, and Figure b shows the flow chart of exam management module.

Fig 11 below shows the flow chart of the learning management and exam management module.

Fig 11A shows the realization of the learning management module. The system realizes this function, the program queries the learning information table according to the acquired learning course course_id, returns the learning course code course_code and the learning course type course_type, and uses the Create and Get methods to create and present corresponding management interface. Fig 11B shows the implementation of the exam management module. The program queries the exam information table according to the acquired exam_id, and returns the exam code exam_code and exam type exam_type, and uses the Create and Get methods to create and present corresponding management interface. The interface presentation, management, and retrieval of these user-selected interfaces are completed by the ProvisionDeviceInfo class calling the service management class InformationManager.

### Performance test of smart system

I. The test environment of the smart classroom teaching system mainly includes the hardware environment of the IoT engineering experiment platform constructed with the wireless switch and its distributed wireless system as the core. In addition, there is the intelligent access gateway and its distributed wired system, and the software environment such as related operating systems and databases. The specific test environment configuration is shown in Table 1.

**Table 1:** Configurations for test environment of smart classroom teaching.

Name	System version
Web application server	CentOS 6.0
Web application language	Java
Web server middleware	apache+Jboss+Jdk1.5 MySQL
Database server	UNIX
Front communication server	Windows Media Services 9 Windows®7 Professional 32-bit WCS2410C
Flow media services	Optional
Workstation	Optional
Optical carrier wireless switch	CentOS 6.0
Smart access gateway	Java
Network hard disk video recorder	apache+Jboss+Jdk1.5 MySQL

II. The system test randomly tests the system from the user’s point of view under the conditions of the user’s actual use of the software to observe whether there is a major failure defect in the system. The Linux+Nginx+tomcat is selected as the cluster server. lvs is used for load balancing and dual-system hot backup to avoid a single point of failure affecting the normal operation of the system. In the event of a failure, the keepalived module is applied to transfer the failover to the backup server. In addition, the MySQL database is processed using the distributed server cluster to reduce the pressure of big data processing.

Students in two classes in a university who are taught by an English teacher are selected for analysis of teaching effect. The two classes have the same number of students, which means 36 students in class A and 36 students in class B. The two classes have the same teaching experiment environment based on the smart classroom to analyze the changes in the performance of the two classes before and after processing.

III. Experiment design

First of all, it needs to arrange two classes as two groups of subjects, the experimental group and the control group, which are not selected according to the principle of randomization. It is necessary to analyze the previous English scores in the grade and interview with teachers according to the realistic characteristics of the quasi-experimental research. Then, two classes with equivalent English proficiency are selected to participate in the experiment, so as to ensure the normal conduct of the experiment. The English proficiency of students in the two classes are checked to provide comparative data and difference analysis for the experimental post-test data. One class is selected as the experimental class, which accept the arrangement of the experimental teaching method from the two determined subject classes. The other class is undertaken as the control class, which is tested according to the conventional teaching plan. Therefore, Class A and Class B in the first grade of a university in Chongqing are mainly analyzed. The reasons for choosing these two classes are summarized as follows. i. The English subjects are divided into four levels based on the comprehensive scores of the middle and end of the English semester. The two classes are in the same grade B in the comprehensive ranking of the last semester according to the English teachers of this grade, and there is a difference of 1 place. ii. The English teachers in these two classes are the same. iii. The numbers of the two classes are equal, with 36 students in class A and 35 students in class B. iv. The two classes have the same teaching experiment environment based on the smart classroom.

The vocabulary post-test is arranged once a week, and twice phased tests are performed. The post-test paper is designed by the English teachers who have undergone the experiment and the English teachers of the same grade group in the school. The test time is set in a self-study class on Friday afternoon of the fourth week. The test time is 20 minutes. The test papers are sent to the students in the experimental class and the control class at the same time. After the test, the test scores of the two classes will be collected and counted. The data obtained from the test is post-test data, the first test score data is post-test 1 experimental data, and the second test score data is post-test 2 experimental data. English vocabulary learning mainly focuses on mastering vocabulary spelling, vocabulary interpretation, and vocabulary use. The test questions mainly include the above three sub-items. The test questions mainly include fill-in-the-blank questions and multiple-choice questions. Fill-in-the-blank questions examine students’ mastery of the basic tenses and voices of vocabulary, the degree of mastery of word meaning and spelling, and multiple-choice questions examine the use of vocabulary in sentences. There are 10 multiple-choice and fill-in-the-blank questions each, each with 1 point and a total score of 20 points. The questionnaire surveys and studies the students’ information literacy and basic abilities, attitudes and methods of English vocabulary learning, knowledge of learning using vocabulary tools, and preference for vocabulary learning resources. The 9 questions are 1–8, and the options of each question are divided into A\B\C\D according to classifications (1 = A, 2 = B, 3 = C, and 4 = D).

All tests are conducted without reminding for review, and invigilated by the participating teachers and the author to ensure the reliability and validity of the experiment. The objective questions of all test papers are automatically corrected by the mobile teaching platform, and all the subjective questions of the test papers are corrected by the author to ensure the fairness of the experimental test. Finally, the 2 post-test data of the experimental class and the control class that participated in the experiment are statistically sorted and analyzed. The first is to sort out the data from the score section of the total test score, analyze the changes in the score level of the students in each test, and analyze the impact of the experiment on the overall level of the class. The second is to count the average score, standard deviation, maximum value, and minimum value of each question type in each test according to the sub-items of the question type. Then, which internal knowledge of the student’s changes during each test is analyzed. The third is to count the average score, standard deviation, maximum value, and minimum value of the sub-item in all tests according to each sub-item of the question, so as to analyze the changes in the learning ability of a specific sub-item mapped by students during the learning process. Finally, interviews are designed according to the shortcomings of the experiment, so as to have a deeper understanding of the inner thoughts of teachers and students, and provide the most cutting-edge data for the experiment.

Two weeks after the experiment, the teacher still adopts this teaching method for vocabulary teaching, and collects the mid-term and final test score data of the class. Then, the results are compared to verify the positive impact of the teaching method on the level of English learning.

## Result analysis

### Performance test of the system

**Fig 12:**
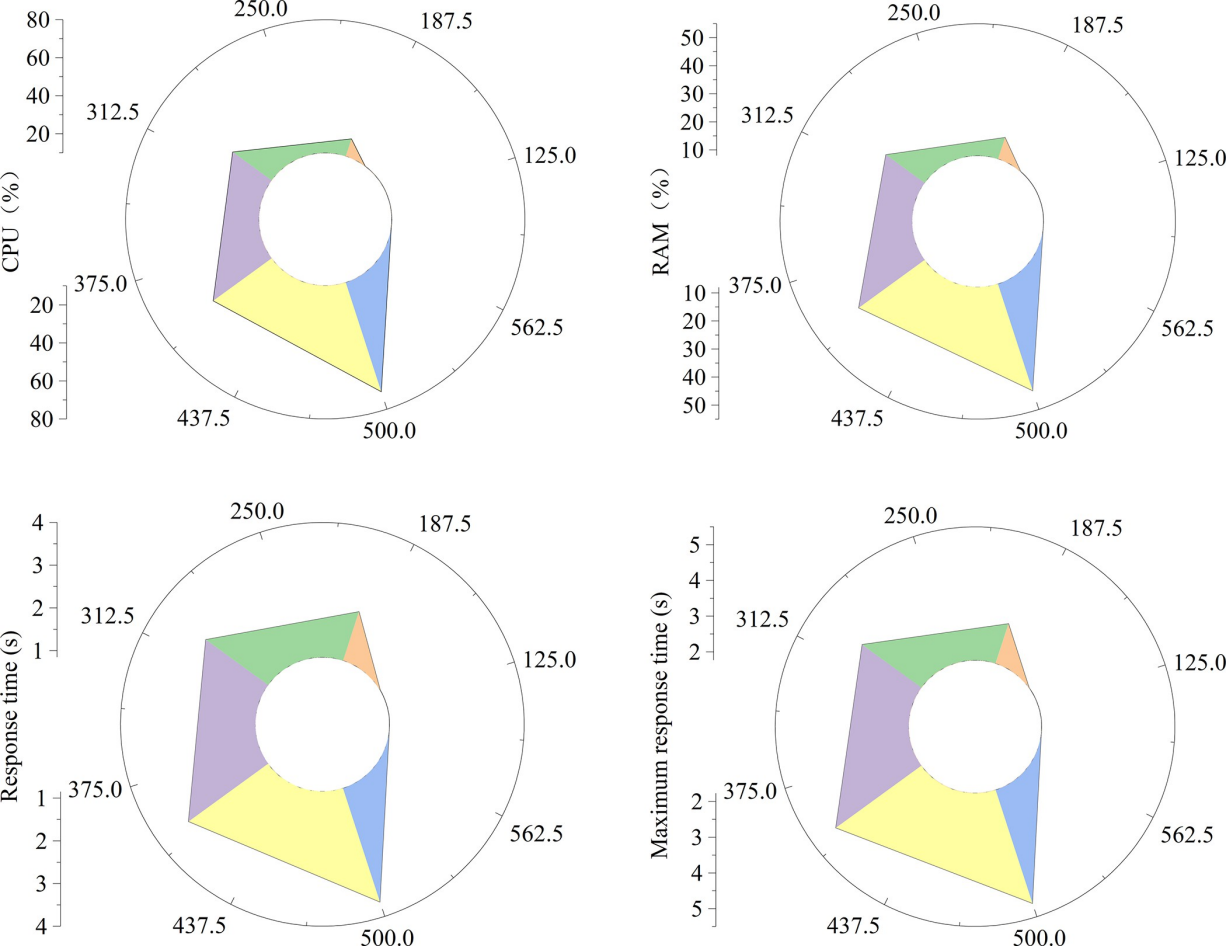
The data graphs for user load test.

From Fig 12A–12D, occupancy rate of memory, average response time, and maximum response time of the system are gradually increasing as the number of users continues to increase, the occupancy rate of CPU. When the number of users reaches 500, the average response time of the system is 3.65 seconds, and the maximum response time is 5.11 seconds, which is in line with the expected values; and the occupancy rates of CPU and memory can also ensure the stability of the system.

**Fig 13:**
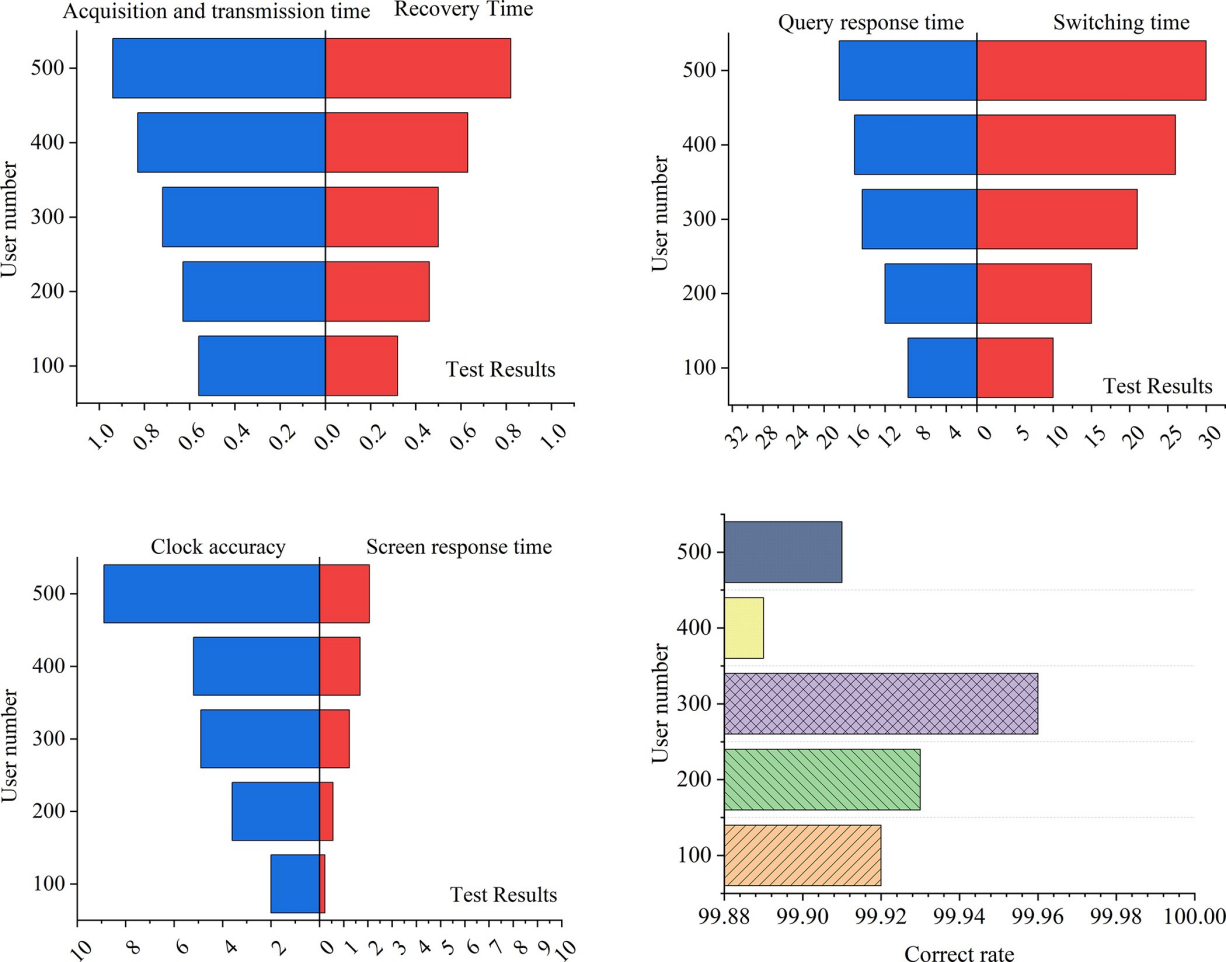
Data results for other performance test of the system.

Fig 13A shows the collection, transmission, and recovery time under different user loads. Fig 13B reveals the query response time and call screen response time under different user loads. Fig 13C gives the switching time between the active and standby machines and the accuracy of the system clock under different user loads. Fig 13D shows the restart recovery time under different user loads. It is found that the performance indicators of the smart English classroom system proposed in this study meet the application requirements of school teaching.

### Comparative analysis of different models

**Fig 14:**
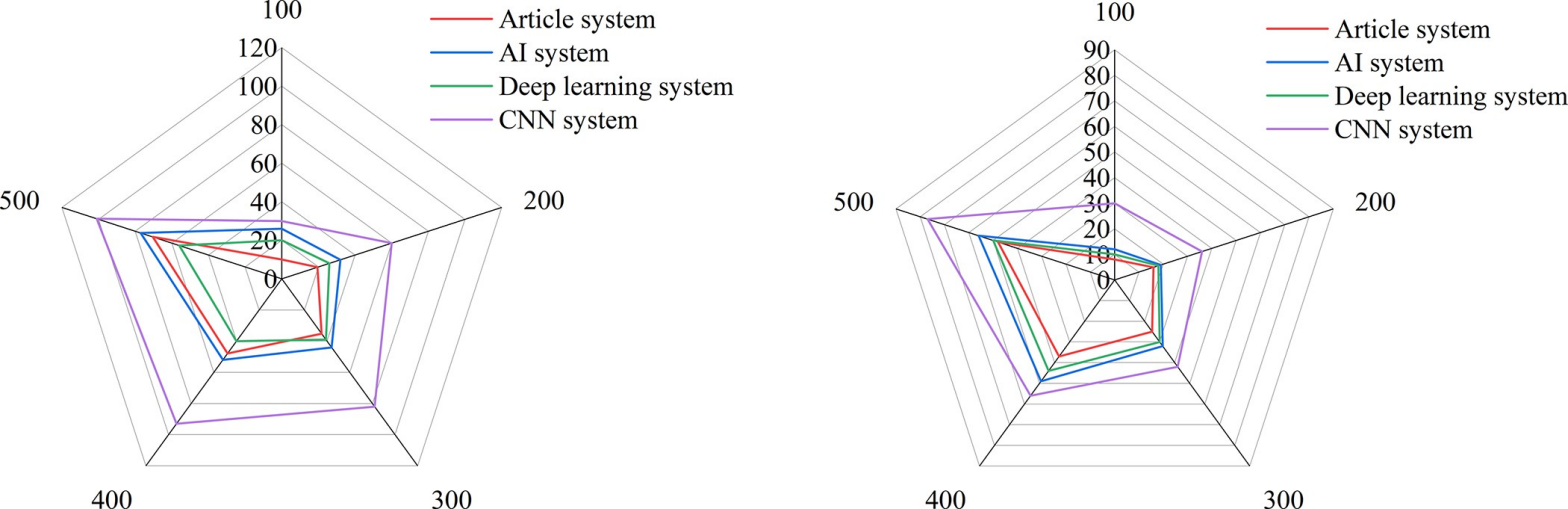
The occupancy of different smart classroom systems.

Fig 14 shows that the model proposed is compared with the models in previously studied. The core of the AI system [28] is constructing the face recognition process, the core of the deep learning system [29] is focusing on the emotions of students, and the core of the convolutional neural network (CNN) system [30] is based on the design atmosphere and audio data to mobilize classroom equipment. Fig 14A shows the occupancy rates of CPU and memory of the models. It is found that the system proposed in this study occupies less CPU. Although the deep learning and AI system occupies less CPU, it requires the cooperation of graphics processing unit (GPU), which greatly increases the cost of teaching.

**Fig 15:**
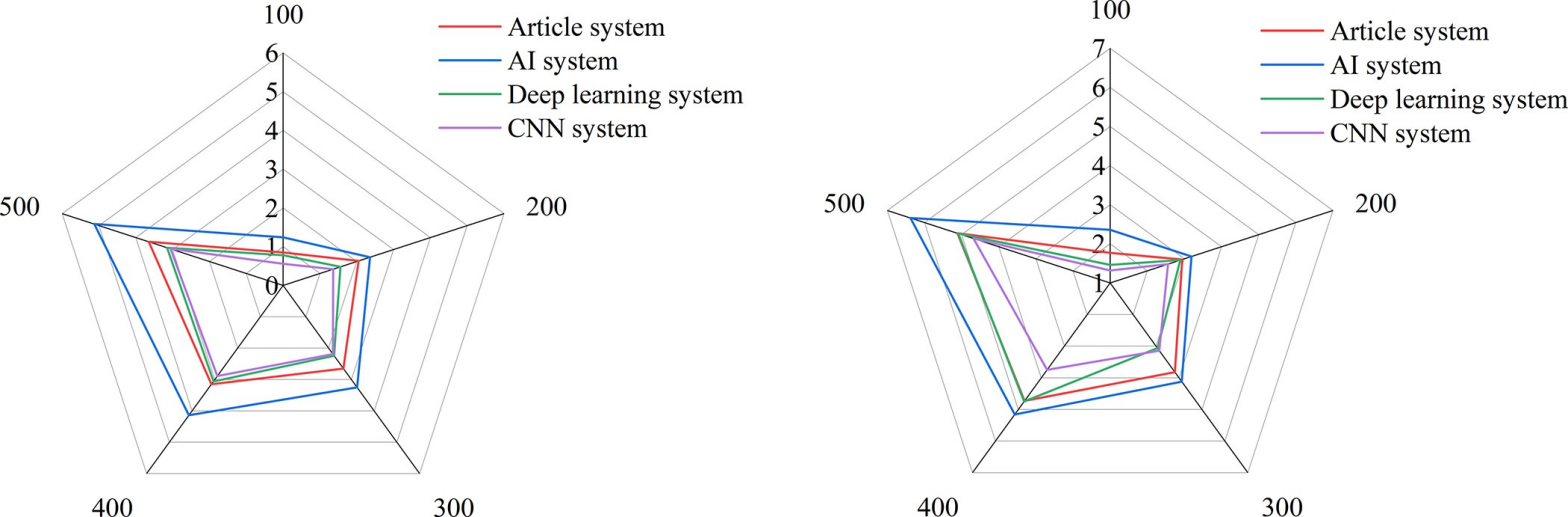
The response times of different smart classroom systems.

Fig 15A shows the average response time of different smart classroom systems, and Fig 15B shows the maximum response time of different smart classroom systems. It is found that the system based on deep learning and CNN have obvious advantages in processing time as the number of users increases, but the average response time of the system proposed in this study is basically maintained at about 3s, which verifies the effectiveness of the proposed smart classroom system.

### Test on teaching effect

**Fig 16:**
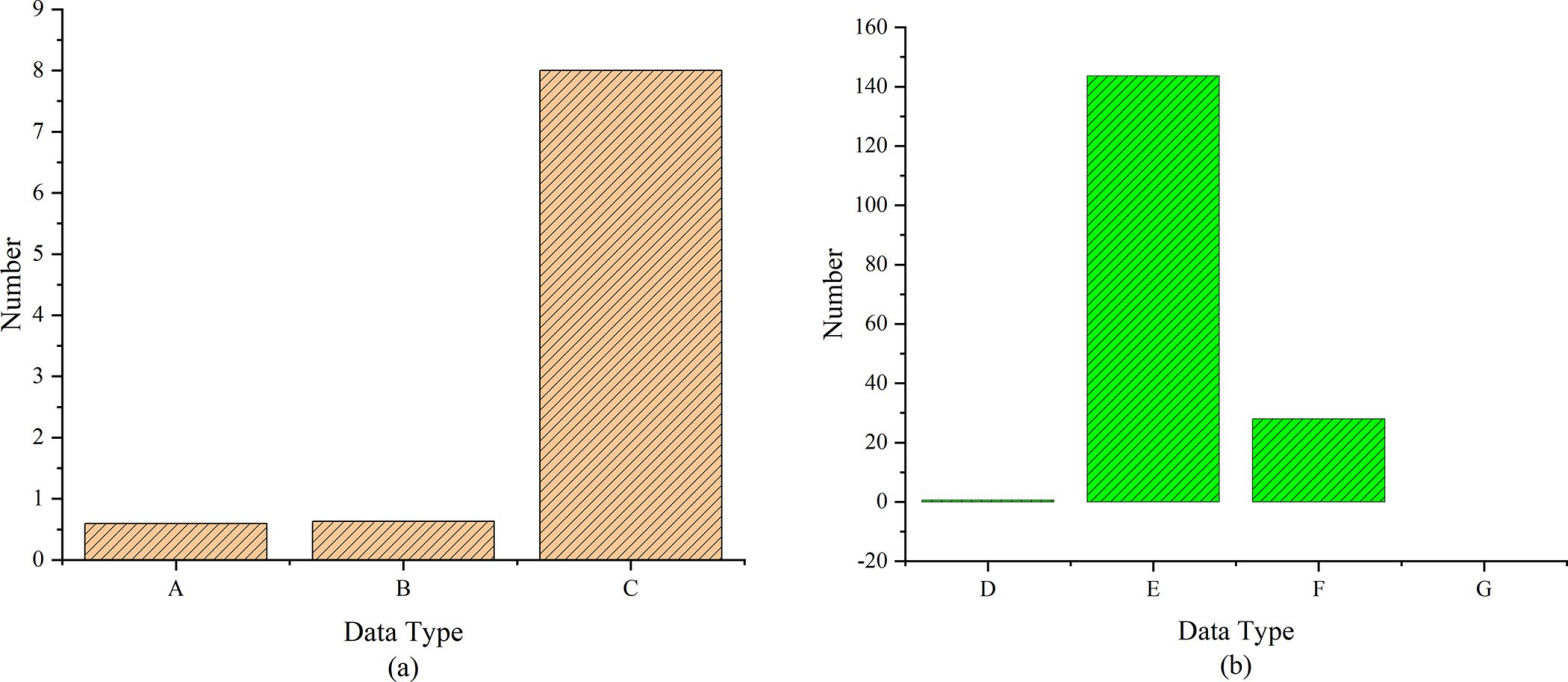
Reliability and validity data statistics of the questionnaire. Note: Figures a and b show the analysis results of reliability and validity, respectively. A ~ F in the figure represent Cronbach’s Alpha, Cronbach Al value based on standardized items, number of items, KMO value, Bartlett’s spherical test, df, and Sig, respectively.

Fig 16 below shows the reliability and validity data statistics of the questionnaire.

**Fig 17:**
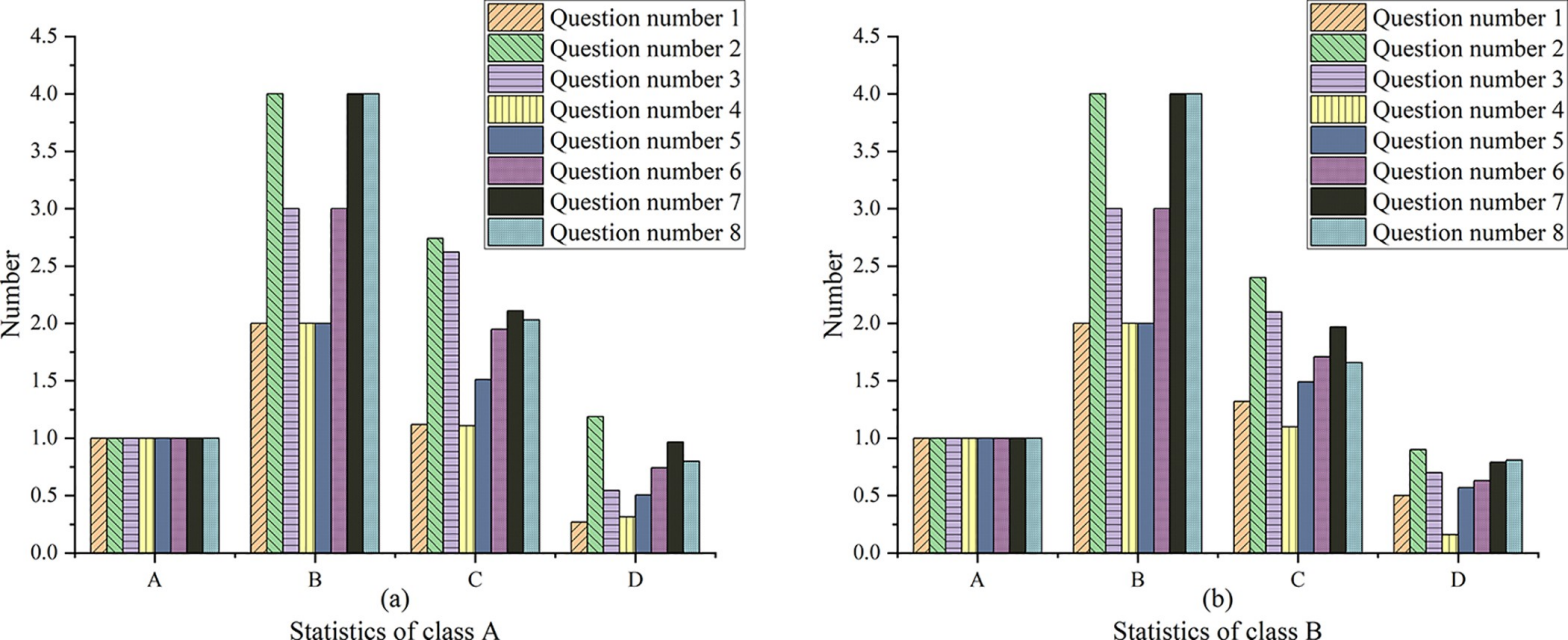
Questionnaire survey data of two classes. Note: A~D in the figure represent minimum value, maximum value, mean value, and standard value, respectively.

The statistics in the figure above illustrate that the Cronbach’s Alpha value is 0.631 > 0.5, indicating that the questionnaire shows good reliability. Factor analysis of the questionnaire suggests that the value of KMO is 0.619 > 0.5, which indicates that the questionnaire shows high validity. Fig 17 below shows the survey data of the two classes.

It is found from the data presentation results in Fig 17 that there is no significant difference between the answers of the students in the experimental class and the control class to the questions in the questionnaire. Students in both classes have basic computer literacy, and the proportion of students using vocabulary learning tools before the experiment is large in the experimental class. However, students use the same frequency as the control class. Students in the experimental class are less likely to make vocabulary learning plans than those in the control class, but students in both classes spent roughly the same amount of time learning English vocabulary, between 10 and 20 minutes. In terms of the attitude of using vocabulary tools to learn vocabulary, the students in both classes show willingness to try. Based on the above data analysis, students in the experimental class and control class have a positive attitude towards using English vocabulary tools for vocabulary learning, and have the corresponding information technology literacy, so experiments can be carried out in the two classes.

## Analysis of examination paper test results

**Fig 18:**
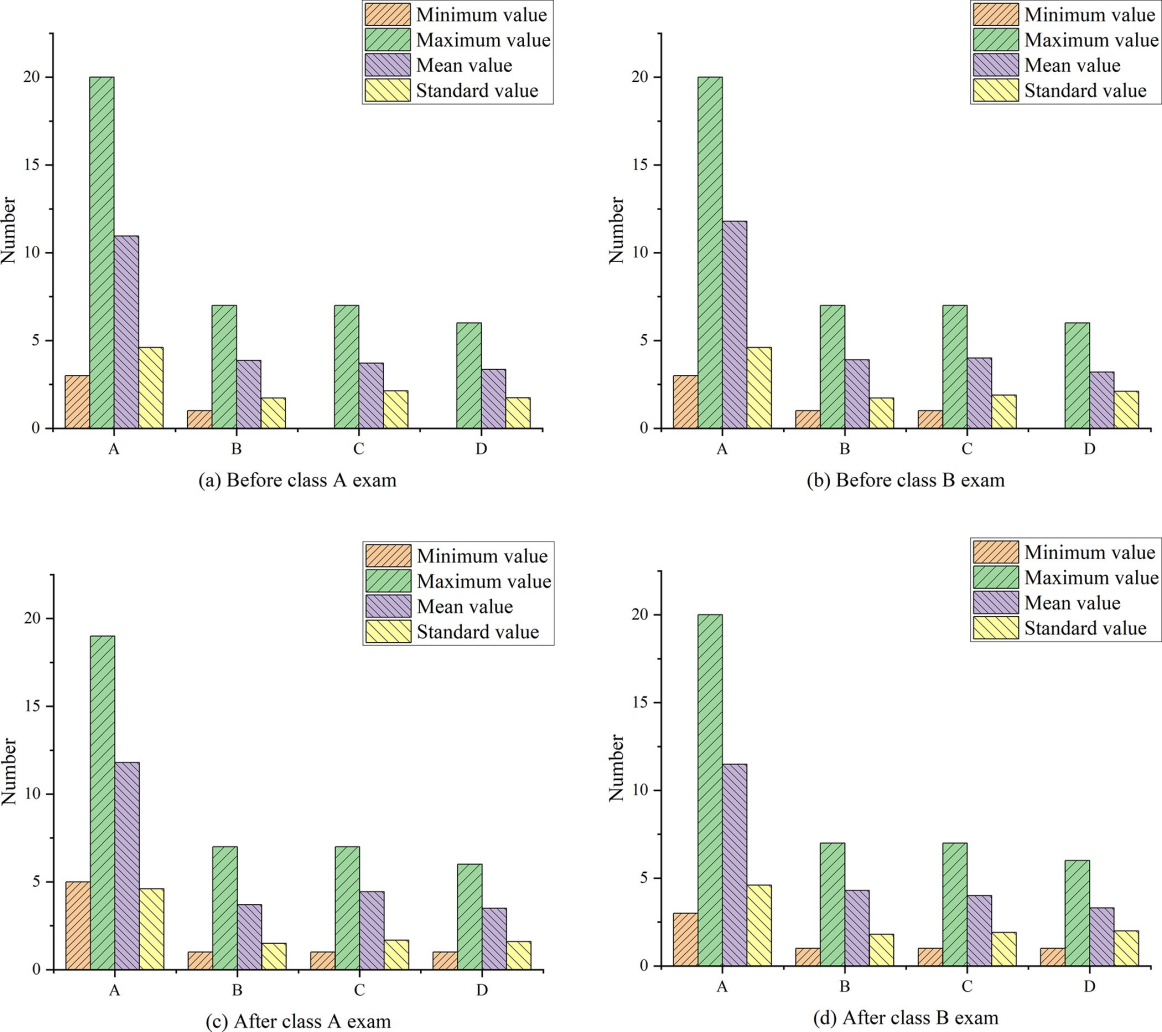
Test paper data statistics before and after the test. Note: A: total score statistics; B: vocabulary spelling questions; C: vocabulary definition questions; D: vocabulary use questions.

Fig 18 below shows the test paper data statistics of the two classes before and after the test.

**Fig 19:**
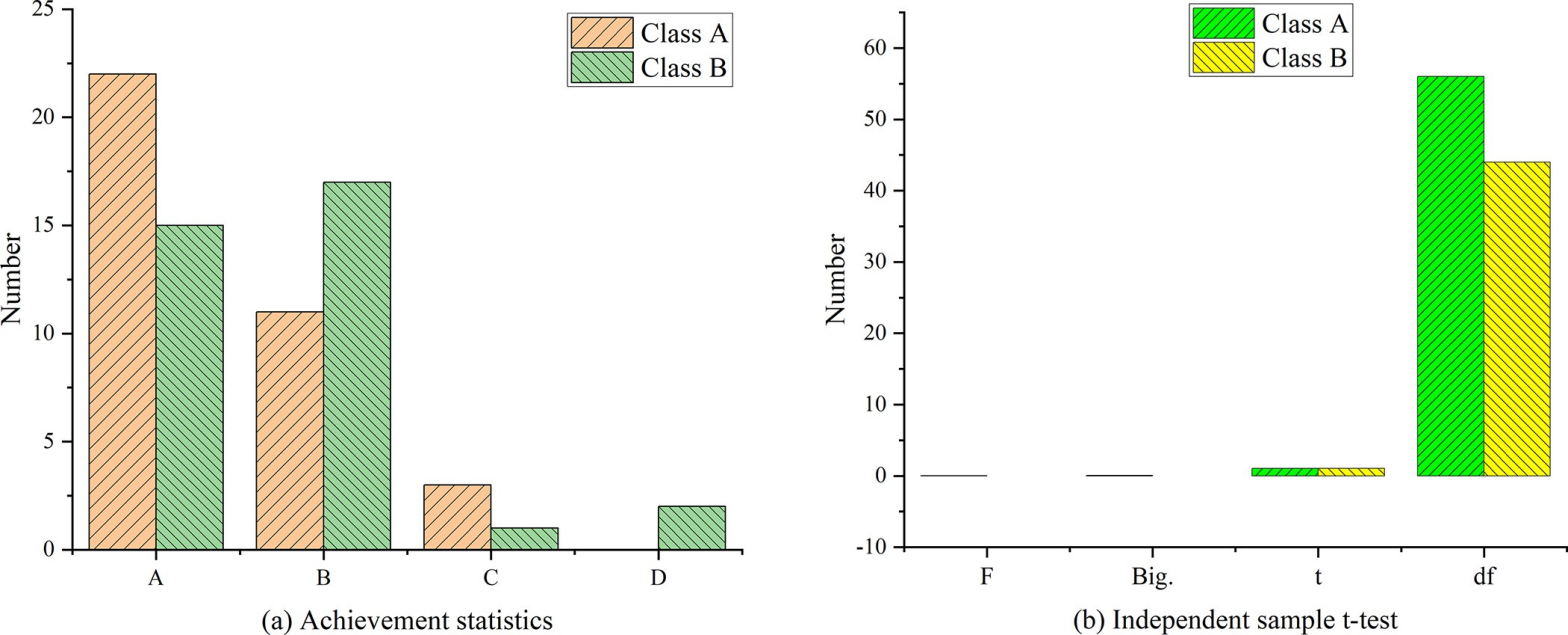
Statistical analysis of mid-term results. Note: A: 120 points—150 points; B: 90 points—119 points; C: 60 points—89 points; D: 0 points—59 points.

Fig 18A and 18B show that in terms of vocabulary spelling questions, the level of the experimental class and the control class is basically similar with little difference. In the vocabulary definition questions, the average score of the control class is higher than that of the experimental class, which indicates that the level of students in the control class is significantly higher than that of the experimental class. In the vocabulary use questions, the average scores of the experimental class and the control class are not much different, but the level of the experimental class is slightly higher than that of the control class. From the average score of the total score, students in the control class have a better grasp of vocabulary than the experimental class. Fig 18C and 18D show that the overall score of the students after taking the test has not improved much in the data statistics of the test paper. There is no significant difference in the scores of vocabulary spelling questions, and the level of learning ability of this type is relatively stable, with a small increase in scores. In the vocabulary definition questions, the overall score of the experimental class is stable while rising, the average score is improved, and the trend of improvement is maintained. Compared with the first post-test, the scores of the students in the experimental class decrease, and the overall gap between the students decreases. Therefore, in the early stage of teacher-assisted vocabulary learning tools, it is helpful to improve students’ learning ability required by vocabulary spelling and reading questions. Fig 19 shows the statistical analysis of middle school results.

**Fig 20:**
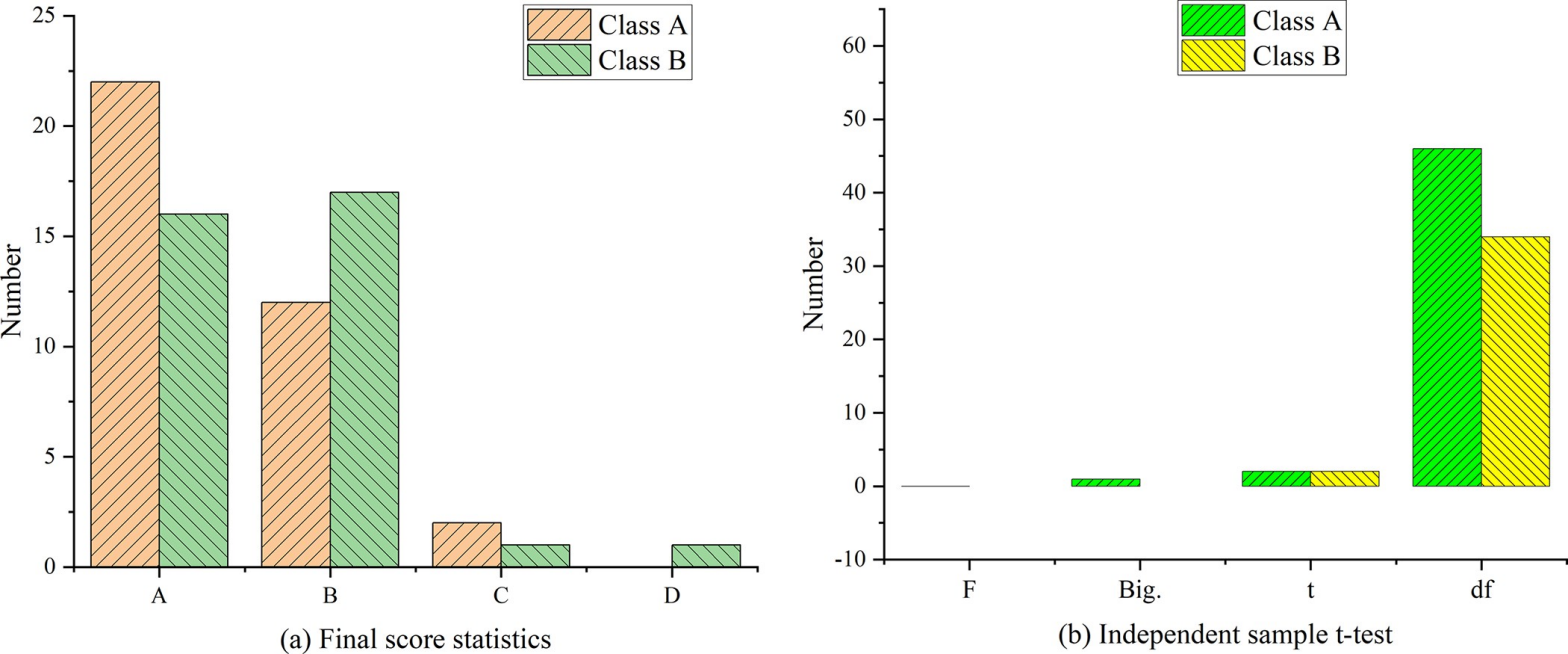
The statistical analysis of final grades. Note: A: 120 points—150 points; B: 90 points—119 points; C: 60 points—89 points; D: 0 points—59 points.

In Fig 19A, from the average score of this mid-term exam, the score of experimental class A is higher than that of control class B. Compared with the test scores before the experiment, the scores of both the experimental class and the control class have improved. The overall score of the experimental class has improved significantly, and the overall score of the experimental class ranks higher than the control class. After half a semester of English vocabulary teaching experiment, it is revealed that the English score of experimental class A increases from 25 to 33 students in 90–150 sections. The number of passing students increases from 70% in last semester to 91% in this semester, and the number of high score (120–150) students increases from 36% to 47%. The number of people with low scores (0–60 points) has decreased significantly, that is, the number of people who failed has decreased significantly compared to last semester, only 8% of people failed. The overall score of experimental class B has been improved and maintained a relatively stable growth. The number of middle and high score students has increased from 27 in the last semester to 32. The number of people passing increases from 77% to 83%, with the number of people in the upper grades (120–150) rising from 40% to 43%. The number of people with lower scores is also shrinking, from 23% to 9% of the total. Therefore, by comparing the scores of the experimental class and the control class, it is found that the passing number of the experimental class A is 8% higher than that of the control class. The number of failed students is 9% lower than that of the control class, and the number of high score students is 4% higher than that of the control class, which verifies the effectiveness of the method in this research. The difference analysis of test score data in Fig 19A results are shown in Fig 19B. *F* value is 0.026, and *P* value is 0.873. If 0.01 < *P* < 0.05, the difference is significant. If *P* < 0.01, the difference is extremely significant. The P value of this study is greater than the significant difference level of 0.05, so it is considered that there is no significant difference in the variance of the difference between the midterm test and the final test of the last semester between the experimental class and the control class. When the variances are equal, the P value of the statistic is 0.308, higher than the significance level of 0.05, so it is considered that there was no significant difference between the experimental class and the control class in the two tests. To sum up, the experimental class and the control class are still at the same level of English learning, and there is no gap in academic performance. However, the performance of the experimental class is significantly improved, while that of the control class is slightly smaller than that of the experimental class. Moreover, the performance of the experimental class changes from lagging behind the control class to overtaking the control class. The above data can prove that the use of this vocabulary learning tool has a positive effect on the overall level of English learning on the condition that it conforms to the classroom teaching schedule. Fig 20 shows statistical analysis of final score.

Fig 20A shows the statistical results of the final grade. According to the average score of the final exam, the score of experimental class A is significantly higher than that of control class B, and the grade level of the two classes also opens up. Compared with the mid-term exam results, the experimental class and the control class have improved, and the overall score of the experimental class has improved significantly. The number of students with intermediate and high scores (90–150 points) in experimental class A reaches 34, which means 97% of the total number of students with passing scores. Among them, the number of high score section (120–150 points) increases from 47% to 61%, and the number of low score section (60–89 points) decreases significantly, that is, only 2 people failed. And the number of people in the 0–59 score segment has steadily decreased to 0. It shows that the method in this research has a significant effect on improving the scores of students with poor English. The difference analysis of the final score data in Fig 20A shows that *F* value is 0.002 and *P* value is 0.0961, which is greater than the significant difference level of 0.05. It is considered that there is no significant difference in the variance of the final test between the experimental class and the control class. When the variance is equal, the *P* value of the *T* statistic is 0.046, which is less than the significant level of 0.05. It is considered that there is a significant difference between the experimental class and the control class in the final test. It is summarized from the difference analysis of the midterm and final scores of the experimental class and the control class that although there is no significant difference between the two classes in the midterm exam, the average score of the experimental class is 6.17 points higher than that of the control class, which is significantly improved compared with post-test 1 and post-test 2. This indicates that the experimental class is in a state of continuous progress, while the average score of the control class is slightly improved, but the range is small. There is a significant difference between the two classes in the final exam scores. According to the results analysis data, it is concluded that the longer the teaching method experiment is conducted, the more significant the progress of students’ scores will be. Therefore, this experiment has a significant promotion effect on the improvement of English learning level.

## Conclusions

This study mainly explores the design of English smart classroom based on the IoT and the research on the practical application effect in college students. Firstly, the English smart classroom system is designed, and the system functions and system composition of the existing smart classroom teaching system platform are analyzed in terms of the structure and performance of the auxiliary teaching equipment terminal, the teaching business process, and the main characteristics. The software modules of the main application functions of the system platform are designed and implemented, and the hardware simulation environment of the software system functions of the English smart classroom teaching system platform is built. Then, a comprehensive test is carried out. In addition, the problems in the test are analyzed, and performance is optimized. After which, a comprehensive platform of smart classroom teaching system is initially constructed with reliable and safe system, wide applicability, and powerful scalability. Secondly, the control group and the experimental group are set up for comparative analysis, and data are collected through questionnaires. The results show that the students in the experimental class have a decline in listening test results compared to the first post-test, and the overall gap among the students has been reduced. Therefore, the vocabulary learning tools assisted by teachers in the early stage of learning can help students to improve their vocabulary spelling and reading questions. Then, English scores of the students in the two classes in the mid-term and at the end of the term are compared. It is found that the two classes have more significant differences in the final exam scores. The data reveals that the longer the teaching method experiment is carried out, the more significant the progress of the students’ scores. Therefore, the experiment has a significant role in promoting the improvement of English learning level. However, there are still some shortcomings due to time and conditions constraints. Firstly, it only conducts research and practice on the main teaching business processes and smart classroom equipment systems of educational units. The content has not been refined in terms of system reliability, security, user experience, intelligent analysis, operation and maintenance management. Secondly, the research on the related technical standards of the smart classroom teaching system, the sharing of high-quality teaching information resources, and the visual operation and maintenance management application of the smart classroom equipment system have not been perfected. In the follow-up, it will analyze deeper from these two perspectives to continuously improve the smart English teaching system.

## Supporting information

S1 Data Figure data and figure source files of all the figures. (RAR)
